# Atypical Metastases from Prostate Cancer: Alpha-Methylacyl-Coenzyme A Racemase (AMACR) as a Potential Molecular Target in Prostate-Specific Membrane Antigen-Negative Prostate Adenocarcinoma

**DOI:** 10.3390/biom15010017

**Published:** 2024-12-26

**Authors:** Ilham Badrane, Angelo Castello, Matteo Brunelli, Corrado Cittanti, Sara Adamantiadis, Ilaria Bagni, Noemi Mindicini, Federica Lancia, Massimo Castellani, Licia Uccelli, Mirco Bartolomei, Luca Urso

**Affiliations:** 1 Department of Translational Medicine, University of Ferrara, Via Aldo Moro 8, 44124 Ferrara, Italy; ilham.badrane@unife.it (I.B.); sara.adamantiadis@unife.it (S.A.); ccl@unife.it (L.U.); rsulcu@unife.it (L.U.); 2 Nuclear Medicine Unit, Onco-Hematology Department, University Hospital of Ferrara, 44124 Ferrara, Italy; m.bartolomei@ospfe.it; 3 Nuclear Medicine Unit, Fondazione IRCCS Ca’ Granda Ospedale Maggiore Policlinico, 20122 Milan, Italy; angelo.castello@policlinico.mi.it (A.C.); massimo.castellani@policlinico.mi.it (M.C.); 4 Department of Pathology and Diagnostic Health, University of Verona, 37100 Verona, Italy; matteo.brunelli@univr.it; 5 Anatomic Pathology Unit, University Hospital of Ferrara, 44124 Ferrara, Italy; i.bagni@ospfe.it; 6 Oncology Unit, University Hospital of Ferrara, 44124 Ferrara, Italy; noemi.mindicini@gmail.com (N.M.); lncfrc@unife.it (F.L.)

**Keywords:** prostate cancer, PCa, PSMA, PSMA PET, PET/CT, PSMA negative PCa, AMACR, alpha-methylacyl-coenzyme A racemase, P504S, atypical metastases

## Abstract

Prostate cancer (PCa) is a high-prevalence disease usually characterized by metastatic spread to the pelvic lymph nodes and bones and the development of visceral metastases only in the late stages of disease. Positron Emission Tomography (PET) plays a key role in the detection of PCa metastases. Several PET radiotracers are used in PCa patients according to the stage and pathological features of the disease, in particular ^68^Ga/^18^F-prostate-specific membrane antigen (PSMA) ligands. Moreover, 2-deoxy-2-[18F]fluoro-D-glucose ^18^F-FDG PET usually shows metastases in the late stages of disease, when dedifferentiated neoplastic clones lose PSMA expression. In some cases, PCa patients may present atypical sites of metastases, with uncommon appearance at PET imaging with different radiotracers. We present the case of a patient with biochemical recurrence of PCa (ISUP Grade Group IV; PSA 4.7 ng/mL) showing atypical sites of metastases (the testis and multiple lung nodules) with absent PSMA expression and high [^18^F]FDG avidity. The patient showed diffuse positivity to alpha-methylacyl-coenzyme A racemase (AMACR). Moreover, a literature review was performed by collecting cases of PCa patients with atypical metastatic spread detected via PET imaging, with the aim of highlighting the relationship between atypical sites of metastases, imaging presentation, and pathology findings.

## 1. Introduction

Prostate cancer (PCa) is the second most prevalent malignancy worldwide, with 1.3 million diagnoses every year and approximately 2.3 million new cases by 2040 [1]. Surgery, robot-assisted or not, and radiation therapy represent the two options for radical treatment in localized PCa, although active surveillance can be considered in selected patients. Nevertheless, as many as 30–50% of PCa patients relapse in the first 10 years of curative-intent therapy [2,3,4,5]. Monitoring serum levels of prostate-specific antigen (PSA) is the historical and still valid biomarker for PCa recurrence, also increasing before imaging-detectable signs by months or even years. This condition is indicated as biochemical recurrence (BCR) and defined as a PSA level above 0.2 ng/mL (0.2 μg/L) across two measures (and rising) after radical prostatectomy or a PSA level equal to or greater than 2.0 ng/mL above the nadir after radiation therapy [6,7]. However, PSA does not allow for the identification of the site of recurrence (i.e., local, regional, or systemic), which is crucial for further patient management. In this context, imaging can provide valuable information and guide specific treatment approaches.

**Figure 1:**
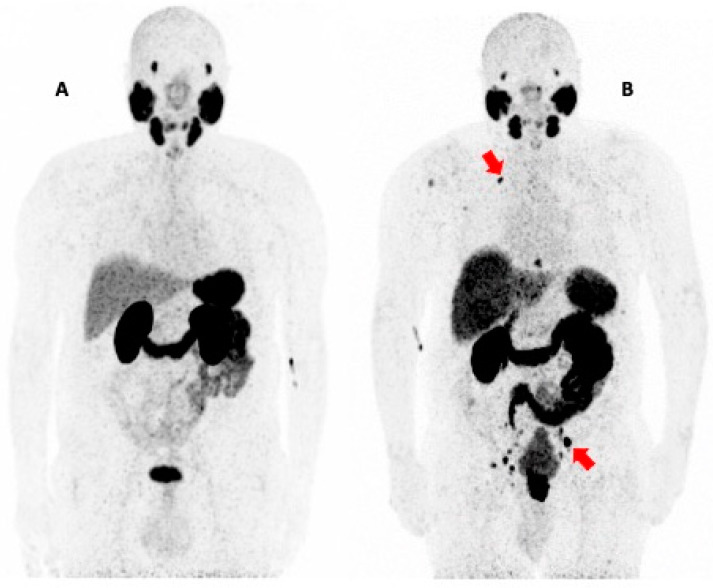
The figure shows the maximum intensity projection (MIP) of 2 [^68^Ga]Ga-PSMA-11 PET/CT studies. The first (**A**) was performed in a patient with BCR of PCa after radical prostatectomy (ISUP Grade Group 3; PSA = 0.63 ng/mL), without evidence of pathological PSMA uptake. The study shows the physiological distribution of PSMA ligand PET, with intense uptake in the salivary and lacrimal glands, spleen, and kidneys and mild radiotracer uptake in the liver and bowel. The second scan (**B**) demonstrates multiple metastases of PCa (red arrows) in a patient undergoing primary staging of very-high-risk PCa (ISUP Grade Group 5; PSA = 43.69 ng/mL).

The most frequent sites of PCa metastases are the bones and regional lymph nodes. However, in the last few decades, the detection of atypical sites of PCa metastases has progressively increased in parallel with the improvement in Positron Emission Tomography (PET) technology [8]. In particular, prostate-specific membrane antigen (PSMA) ligand PET imaging has revolutionized the diagnostic management of PCa patients, enabling the detection of a large number of metastases undetectable with conventional imaging modalities, even in atypical sites [9,10] (Figure 1). Recently, evidence of PSMA-negative and 2-deoxy-2-[18F]fluoro-D-glucose ([^18^F]FDG)-positive PCa metastases have been described, in particular in metastatic castration-resistant prostate cancer (mCRPCa) [11,12]. Therefore, multi-tracer PET imaging is progressively gaining relevance in PCa patients [13]. Prolonged overall survival due to the availability of novel therapeutic drugs has increased the number of mCRPC patients and, as a consequence, the detection of atypical patterns of metastatic disease [14,15].

In this article, we describe a clinical case of PCa with atypical sites of metastases and positivity on multi-tracer PET imaging. Moreover, we provide a summary of atypical metastatic patterns reported in the literature.

## 2. Case Presentation

**Figure 2:**
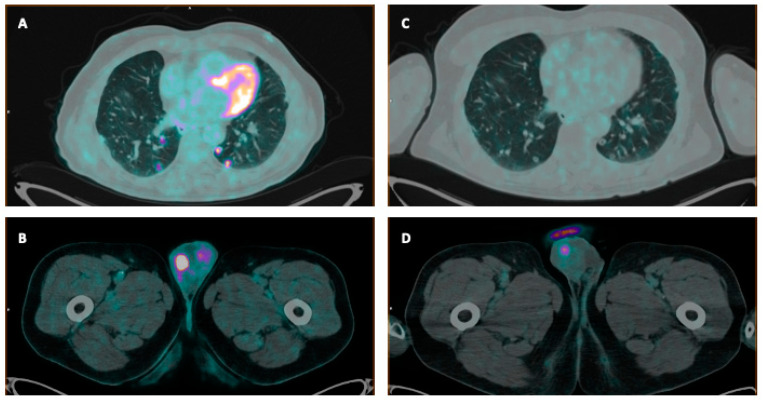
The figure shows axial fused images of [^18^F]FDG (**A**,**B**) and [^68^Ga]Ga-PSMA-11 PET/CT (**C**,**D**). Of note, the right testicular lesion shows much higher uptake intensity at [^18^F]FDG PET/CT than at [^68^Ga]Ga-PSMA-11 PET/CT. Similarly, the lung lesions are [^18^F]FDG avid and PSMA negative. The case highlights an atypical metastatic pattern, both in terms of atypical localizations (testis and lung metastases) and of multimodal PET imaging positivity ([^18^F]FDG avid and with low/absent PSMA expression).

A 72-year-old man with a history of resection of NSCLC (pT2N0) in complete remission for 8 years underwent a radical prostatectomy (RP) and pelvic lymphadenectomy for high-risk PCa. PSA at surgery was 41 ng/mL. Histological examination of the surgical specimen revealed PCa (ISUP Grade Group 5), predominantly located in the left lobe. Additionally, extraprostatic extension, diffuse perineural invasion, and microscopic tumor residuals at the surgical margin were demonstrated. However, the twenty lymph nodes removed did not show metastatic involvement (pT3a N0, R1). Given the anatomic–pathological findings and the dosable PSA levels after surgery (0.24 ng/mL), prostate bed radiation therapy and androgen deprivation therapy (ADT) were recommended. However, the patient refused ADT and only salvage radiotherapy was conducted. The patient was lost at follow-up for 4 years and only recently showed up at the oncology department, with a chest high-resolution CT (HRCT) demonstrating multiple bilateral pulmonary nodules. Alongside, evidence of a rising PSA value (4.7 ng/mL) was detected. Consequently, [^68^Ga]Ga-PSMA-11 PET/CT was performed, showing only a faint uptake in the right testis. A second PET/CT using the glycomimetic tracer [^18^F]FDG was conducted within a month of the previous scan and revealed multiple foci of intense tracer uptake in the pulmonary nodules. Moreover, there was marked uptake in a bilobated area in the right testis (SUVmax 42), along with tracer uptake in some ipsilateral inguinal lymph nodes being detected (Figure 2). Ultrasounds confirmed the presence of a right testicular solid mass.

**Figure 3:**
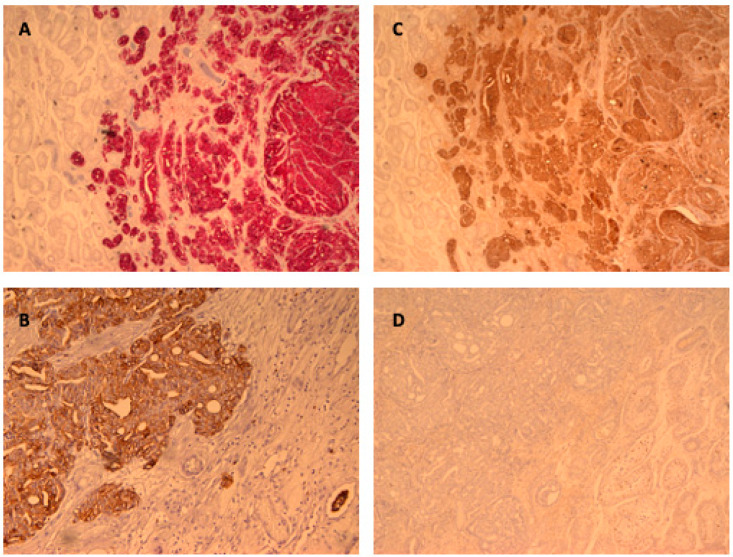
Immunohistochemistry of the resected testicular mass. The mass shows intense positivity to AMACR (**A**), positivity to PCT (**B**) and PSA (**C**), and negative expression for PSMA (**D**).

The case was discussed by a multidisciplinary panel. Based on the PET findings, characterized by high glucose avidity and the relative absence of PSMA expression, relapse of the NSCLC was hypothesized, or primary testicular cancer, with lymph nodes and pulmonary metastases. Subsequently, the patient underwent a fibrobronchoscopy, which was negative, and a right orchiectomy. The histological evaluation demonstrated a testicular PCa metastasis with immunohistochemistry positivity to PSA, diffuse alpha-methylacyl-coenzyme A racemase (AMACR) pan-cytocheratines (PCT), and negativity to PSMA and Sal-like protein 4 (SALL4) (Figure 3). No signs of neuroendocrine differentiation were detected. Given the diagnosis of metastatic castration-sensitive prostate cancer (mCSPC), the patient started ADT plus apalutamide and showed a rapid and remarkable decrease in PSA levels.

## 3. Literature Review

We conducted a literature review investigating atypical sites of metastatic spread of PCa identified using PET imaging, encompassing studies published up to 31 July 2024. The search was executed using the following string: ((prostate cancer[Text Word]) AND (((psma[Text Word]) OR (fdg[Text Word])) OR (pet[Text Word]))) AND ((((uncommon[Text Word]) OR (atypical[Text Word])) OR (unexpected[Text Word])) AND (metastas*[Text Word])). A systematic literature search was performed in the MEDLINE, PubMed, and EMBASE databases. The study focused exclusively on English-language studies, excluding papers not pertinent to the topic. The data retrieved are listed in Table 1.

### 3.1. Genitourinary Tract Metastases

Metastases from PCa to the genitourinary tract are uncommon, and only a small number of cases have been reported in the literature so far. Specifically, we found three cases of penile, four cases of testicular, and one case of simultaneous penile and scrotal involvement, as well as a case affecting the vas deferens.

Despite its rich vascular supply, metastatic spread to the penis is uncommon. Tatkovic et al. [16] reported that the incidence of penile metastasis from PCa was only 0.1% among 4860 [^68^Ga]Ga-PSMA-11 PET/CT scans. This condition typically occurs in advanced stages of the disease, often alongside other common metastatic sites [15,16,17]. However, it could be an isolated metastasis in BCR disease [18]. Detecting metastases in this site is particularly challenging due to the frequent radio-urinary residue in the urethra. Caputo et al. [18] reported a case of a 75-year-old man with a history of ISUP Grade Group 5 PCa, previously treated with external beam radiation therapy and ADT. The patient underwent a [^68^Ga]Ga-PSMA-11 PET/CT scan due to BCR with remarkable PSA velocity (from 0.74 to 4.86 ng/mL in six months). Initially, the scan was reported as negative. Upon review, due to the progressive elevation of PSA, suspicious uptake in the penile shaft and glans, initially misinterpreted as urinary residue, was identified. Yamaga et al. [15] and Mütvelizade et al. [17] reported two cases with similar localizations on a [^68^Ga]Ga-PSMA-11 PET/CT scan but in an advanced stage of the disease. The first case was an 86-year-old man with metastatic PCa (ISUP Grade Group 5, PSA 3.7 ng/mL) involving the bones, liver, and pelvic and retroperitoneal lymph nodes. After RP, multiple non-tender nodules involving the glans and the shaft of the penis upon physical examination were detected. The second case reports a 72-year-old PCa patient (ISUP Grade Group 5) who had significant PSA progression (22.6 ng/mL and 47.9 ng/mL, respectively) despite eight cycles of chemotherapy and enzalutamide treatment. [^68^Ga]Ga-PSMA-11 PET/CT revealed a prostate mass with rectal and bladder invasion and multiple lymph node localizations in addition to an area of intense uptake in the penile shaft. A double localization in the genitourinary tract, respectively to the glans of the penis and scrotum, was reported by Seniaray et al. [19] using [^68^Ga]Ga-PSMA-11 PET/CT in a 68-year-old man on ADT, after transurethral resection of PCa (ISUP Grade Group 4) and radiotherapy. The scan also showed PSMA uptake in the prostatic bed region and in pelvic and inguinal lymph node metastases.

Gupta et al. [20] evaluated the incidence of testis metastases in 1860 [^68^Ga]Ga-PSMA-11 PET/CT scans, identifying three cases. The first case involved a 62-year-old male with a history of PCa (ISUP Grade Group 5, PSA 11.5 ng/mL). Due to the onset of hematuria, urination discomfort, and a PSA level of 13.2 ng/mL, a [^68^Ga]Ga-PSMA-11 PET/CT was performed. The scan revealed a PSMA-avid lesion in the prostate with infiltration into the bilateral seminal vesicles, the posterior wall of the urinary bladder, and the anterior rectal wall. Focal PSMA uptake was also observed in the right testis and the fifth lumbar vertebra. The second case refers to a 77-year-old male with a recent diagnosis of very-high-risk PCa (ISUP Grade Group 5 and PSA levels of 79.38 ng/mL). He was staged with [^68^Ga]Ga-PSMA-11 PET/CT to identify potential metastatic sites. The scan revealed a PSMA-avid lesion in the prostate involving the bilateral seminal vesicles, a pelvic lymph node, and focal PSMA uptake in the right testis. Additionally, mild focal PSMA uptake was noted in the right iliac bone. The third case involves a 69-year-old male who experienced a second BCR with a PSA level of 3 ng/mL after multiple previous treatments, including surgery, hormonal therapy, and radiotherapy. A [^68^Ga]Ga-PSMA-11 PET/CT scan revealed a focal PSMA-avid lesion in the left testis. A subsequent scrotal ultrasound confirmed the presence of a hypoechoic lesion in the left testis. Two similar cases were reported by Yamaga et al. [15] involving a 68-year-old man and an 88-year-old man, both of whom presented with BCR after radical prostatectomy. Their recent PSA levels were 1.2 ng/mL and 21.0 ng/mL, respectively. In both cases, [^68^Ga]Ga-PSMA-11 PET/CT scans revealed a single focus of PSMA uptake in the left and right testis, respectively.

### 3.2. Abdominal Metastases

Peritoneal carcinomatosis is a rare site of secondary localization in PCa due to diffusion from the primary tumor, hematogenous/lymphangitic spread, or iatrogenic seeding during surgery. Only a few cases have been reported in the literature [21,22,23,24]. Simsek et al. [21] documented a case with multiple peritoneal nodules associated with abdominopelvic lymph nodes, along with prostatic bed recurrence, while Zhao and colleagues [22] showed two isolated lesions with intense [^68^Ga]Ga-PSMA-11 uptake. On the other hand, Le Thiec et al. [23] reported two peritoneal metastases in a patient with a very low PSA value (e.g., 0.5 ng/mL), previously treated with surgery and salvage radiation therapy. Histological and immunohistochemical analyses of peritoneal lesions confirmed the recurrence of PCa, with possible dissemination from the primary tumor along the urachus tractus being hypothesized. In addition, Castello et al. [24] showed diffuse malignant [^68^Ga]Ga-PSMA-11 accumulation, consistent with omental infiltration, in a patient referred for restaging with negative [^11^C]-Choline even though they had a PSA level of 1.45 ng/mL. This case confirms the superiority of [^68^Ga]Ga-PSMA-11 over [^11^C]-Choline PET in the detection of peritoneal carcinomatosis.

**Figure 4:**
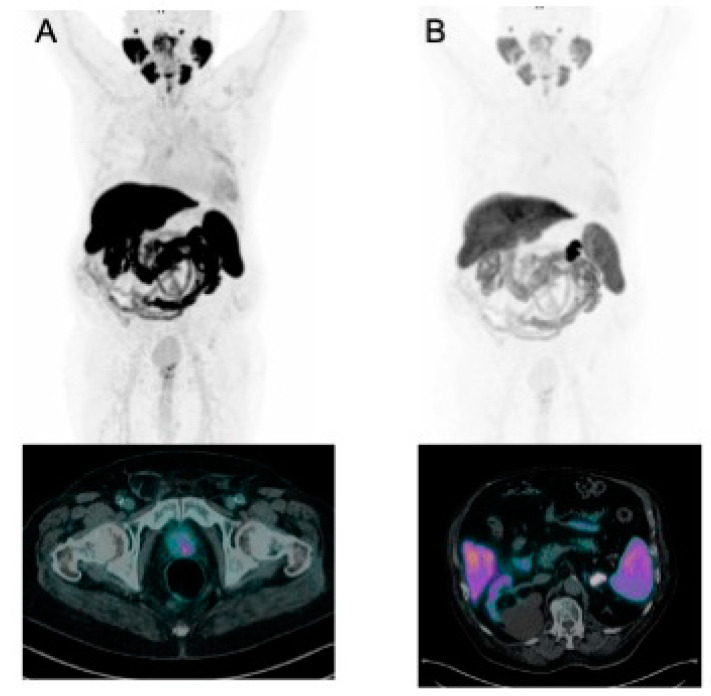
[^18^F]F-PSMA-1007 PET/CT images revealed, along with primary PCa (**A**), a left adrenal lesion (**B**) in a patient with PCa (ISUP Grade Group = 5) during ADT.

Despite adrenal metastases being mostly derived from lung and breast cancer, sporadic case reports with adrenal involvement from PCa have been reported in the literature [17,25,26,27,28] (Figure 4). Initially, Matrone et al. [25] described a case of adrenal metastasis using [^18^F]F-Choline PET with a rapid PSA decrease, from 106.8 to 0.76 ng/mL, one month after adrenalectomy. Moreover, Zhao et al. [28] showed bilateral adrenal lesions with elevated [^68^Ga]Ga-PSMA-11 uptake and no other suspected metastases. After docetaxel chemotherapy associated with ADT, the PSA level decreased and MRI demonstrated a reduction in size. More recently, Üstün et al. [26] aimed to differentiate benign from malignant adrenal lesions detected on [^68^Ga]Ga-PSMA-11 PET/CT scans in 23 patients out of a cohort of 1450 PCa patients. Of note, a SUVmax cutoff value > 6.8 provided both sensitivity and specificity of 100% to distinguish the two groups. Yet, Evbuomwan et al. [27] demonstrated the efficient response of adrenal metastasis after four cycles of ^177^Lu-PSMA RLT in a young 58-year-old African male with aggressive PCa (ISUP 5).

Recently, rectal metastasis is another site that has been added to the unusual localizations from PCa [15,29,30]. For example, Segal et al. [29] described a submucosal rectal nodule on a [^18^F]F-DCFPyL PET/CT scan in a 72-year-old man with biochemically recurrent PCa. Direct invasion through the Denonvilliers fascia, common lymphatic drainage pathways, or tumor seeding in the course of ultrasound-guided transrectal biopsy are the main hypotheses for rectal metastases.

The pancreas is another atypical site of metastasis from PCa. Desai et al. [31] illustrated, for the first time, pancreatic metastasis with [^18^F]-FDG PET/CT, in a 57-year-old male with PCa (ISUP 5) who initially underwent RP, followed by ADT and further treatment with docetaxel after the development of mCRPC.

Despite the liver being a typical site of visceral metastases from PCa, solitary hepatic localization is extremely rare. Önner et al. [32] documented a PSMA-avid liver lesion in a patient who underwent [^68^Ga]Ga-PSMA-11 to stage high-risk PCa. Likewise, Kranzbühler et al. [33] showed, using [^68^Ga]Ga-PSMA-11 PET/MR, a single avid hepatic lesion, located in the falciform ligament, in a patient referred for restaging due to a continuous increase in PSA and negative PET imaging with [^68^Ga]Ga-bombesin and [^18^F]F-choline.

Metastatic involvement of the perirenal fascia has been reported in melanoma, urinary bladder, lung, and gastrointestinal neoplasms, while only Chandekar and colleagues [34] have reported a case from PCa detected with [^68^Ga]Ga-PSMA in a patient with a PSA of 226 ng/mL and ISUP Grade Group 3. As mentioned above, in the same clinical case, Evbuomwan et al. [27] also demonstrated the complete response of a renal lesion after four cycles of ^177^Lu-PSMA.

### 3.3. Toracic Metastases

Cardiac metastases are infrequent in PCa as well as in other malignancies [35]. In the literature, a few cases have been described. The absence of physiological cardiac uptake upon PSMA ligand PET as well as upon [^18^F]F-choline and [^18^F]F-fluciclovine PET guarantees an optimal target-to-background ratio (TBR). Moreau et al. [36] reported, along with numerous typical metastases, a rare localization in the pericardial recess using [^68^Ga]Ga-PSMA-11 PET to stage an 88-year-old man with high-risk PCa (ISUP 5, PSA 66 ng/mL). Previously, another case of interatrial septum metastasis was reported by an Italian group using [^18^F]F-PSMA-1007 PET after the second progression in an mCRPC patient treated with enzalutamide plus ADT [37]. In addition, Das and Yeh [38] illustrated a [^68^Ga]Ga-PSMA-11 metastasis in the right atrium, subsequently confirmed by endomyocardial biopsy, in a 78-year-old man with PCa ISUP 4 (PCa) initially treated with external beam radiation therapy and imaged for BCR due to serum PSA of 2.5 ng/mL. Despite these findings, they did not change the patients’ management due to the extensive disease volume; these cases highlight the capability of PSMA PET radiotracers to detect atypical lesions which would have been probably overlooked by conventional imaging.

Interestingly, Perez et al. [39] described a case of a patient with a history of PCa, with two isolated lung nodules detected using [^68^Ga]Ga-PSMA-11 PET and characterized by different uptake intensities (i.e., low and moderate). Therefore, the patient underwent [^18^F]FDG PET, which showed elevated glucose metabolism for the low PSMA nodule and low metabolism for the nodule with moderate PSMA expression. However, fine needle aspiration confirmed the PCa origin, excluding primary lung cancer. After starting ADT, a second [^18^F]FDG PET showed a response for both lung nodules, demonstrating metabolic heterogeneity from two PCa pulmonary nodules, both responding to treatment.

Yamaga et al. [15] confirmed, through CT-guided biopsy, an atypical site of PCa recurrence in mediastinal lymph nodes in a 70-year-old PCa patient five years after RP (ISUP 5) and three years after radiation therapy and ADT. Although bone metastases are common in PCa, sphenoid bone lesions, associated with involvement of the temporal lobe, maxillary sinus, and orbit as described by Mütevelizade and colleagues in an 84-year-old man who complained of diplopia and proptosis, are quite rare [17]. As a matter of fact, the patient was treated with 7400 MBq of [^177^Lu]Lu-PSMA without any side effects.

### 3.4. Intracranial Metastases

**Figure 5:**
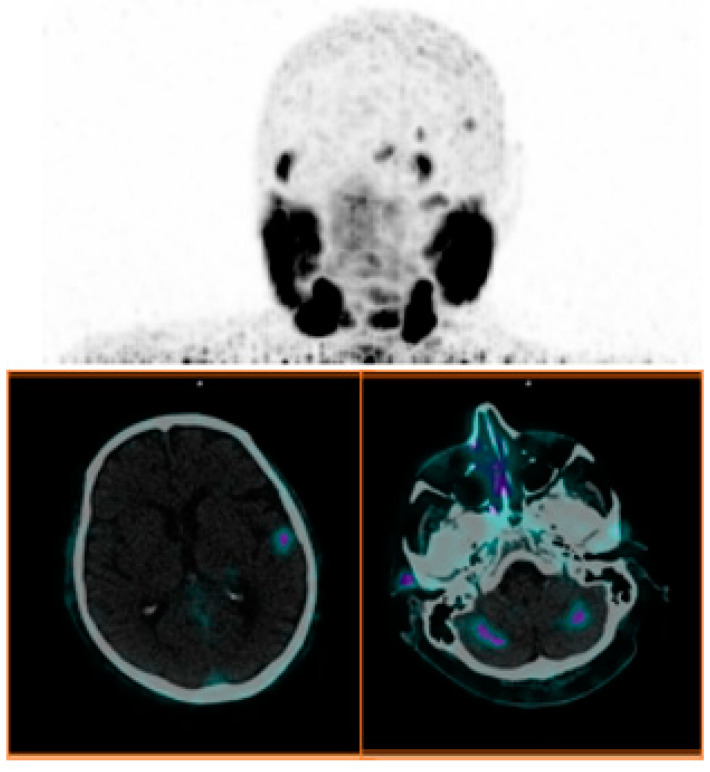
Maximum intensity projection (MIP) and axial fused [^68^Ga]Ga-PSMA-11 PET/CT images in a patient with cerebral and cerebellar metastases in PCa (ISUP Grade Group = 5).

A few reports of brain metastases in PCa have been reported in the literature (Figure 5). For the first time, in a large retrospective analysis of 4341 PCa patients who underwent [^68^Ga]Ga-PSMA-11 PET, McBean et al. [40] demonstrated that the incidence of intracranial metastases was very rare, with it being only 0.18%. Of note, all lesions were in patients with ISUP Grade Group 5 and extensive metastatic disease. Moreover, five out of eight patients had small-cell neuroendocrine variant carcinoma, a de-differentiated PCa well known for being aggressive and generating metastases in atypical location [41].

Likewise, Ajit et al. [42] described a case of a 67-year-old man with PCa with a history of PCa (ISUP 5) initially treated with radical radiation therapy. After the last BCR, the patient also complained of headache. In fact, a subsequent [^68^Ga]Ga-PSMA-11 PET scan detected a metastatic lesion in the left cerebellar hemisphere. The patient underwent palliative whole-brain radiotherapy of 10 fractions and a total dose of 30 Gy, followed by a reduction in PSA levels to 0.1 ng/mL.

On the other hand, Kulkarni et al. [43] revealed an extra-axial mass along the right trigeminal nerve. Initially suggestive of neurinoma, the subsequent histology revealed the prostatic origin, as also demonstrated by elevated levels of serum PSA (130 ng/mL).

Recently, in the era of RLT with [^177^Lu]Lu-PSMA, Al-Ibraheem et al. [44] demonstrated, through SPECT/CT after the last cycle, the appearance of an uncommon metastatic lesion in the right temporal brain lobe, emphasizing the role of post-treatment scintigraphy not only for dosimetry reasons.

### 3.5. Soft Tissue Metastases

PCa metastases to the soft tissues, such as muscles and skin, are very uncommon. Patients who develop such metastatic patterns usually exhibit noticeable symptoms. Skin involvement is an uncommon site for PCa spread, occurring in only 0.06–0.3% of cases [45,46]. These lesions most often present as multiple rubbery nodules or plaques, less frequently as a single nodule, and rarely as edema or a non-specific rash on the skin. Skin metastases in PCa can occur in the pubic or genital area, although distant involvement has also been described. Sivrikoz et al. [47] reported a case of a 70-year-old man with a history of PCa (ISUP Grade Group 5) who presented with skin nodules on the pubic region and swelling in the lower extremities that had appeared over a few months. His PSA value was 755 ng/mL. Increased [^68^Ga]Ga-PSMA-11 uptake corresponded to most of the cutaneous lesions and edema in the left lower extremity, in addition to multiple pelvic and retroperitoneal lymph node metastases. Al-Ibraheem et al. [44] described the case of a 68-year-old man with metastatic castration-resistant PCa and multiple skeletal metastases, eligible for radioligand therapy (RLT). His PSA value was 44 ng/mL. After his final cycle of [^177^Lu]Lu-PSMA, post-treatment SPECT-CT suggested disease progression with uptake in the right temporal brain lobe and left triceps muscle, which was confirmed by a subsequent [^68^Ga]Ga-PSMA-11 PET/CT. During treatment, his PSA levels ranged from 16 to 11 ng/mL without significant fluctuations. Arora et al. [48], on the other hand, described a 75-year-old man with prostate adenocarcinoma (ISUP Grade Group 3) and a baseline PSA level of 140 ng/mL. In addition to the primary lesion, a focal PSMA-avid soft tissue deposit was detected on a PET/CT scan in the right inguinal canal. Metastasis to the inguinal canal is an uncommon finding, though it has been reported in ovarian carcinomas, mesothelioma, and adenocarcinomas of the appendix, pancreas, and rectum. It is believed that scrotal metastasis may spread via lymphatic channels that pass through the inguinal canal.

**Table 1:** Literature review regarding atypical metastatic spread in PCa patients, detected via PET imaging.

**Author**	**Year**	**Disease Setting**	**PSA** **ng/mL**	**ISUP**	**PET Tracer**	**Metastasis Site**
Yamaga et al. [14]	2021	mPC/BCR	3.7–1.2–21.0	5	[^68^Ga]Ga-PSMA-11	Penis and testis (n.2)
Mütevelizade et al. [16]	2022	mPC	3.7–47.9	5	[^68^Ga]Ga-PSMA-11	Brain, adrenal, penis, and orbital
Caputo et al. [17]	2023	BCR	4.86	5	[^68^Ga]Ga-PSMA-11	Penis
Seniaray et al. [18]	2019	BCR	4.86	4	[^68^Ga]Ga-PSMA-11	Penio-scrotal
Gupta et al. [19]	2021	BCR (n.2)/staging	11.5–79.4–3	5	[^68^Ga]Ga-PSMA-11	Testis
Simsek et al. [20]	2020	BCR	104	5	[^68^Ga]Ga-PSMA-11	Peritoneal
Zhao et al. [21]	2023	NA	1.25	3	[^68^Ga]Ga-PSMA-11	Peritoneal
Le Thiec et al. [22]	2019	BCR	0.5	2	[^68^Ga]Ga-PSMA-11	Peritoneal and urachus
Castello et al. [23]	2021	BCR	1.45	5	[^68^Ga]Ga-PSMA-11	Omental
Matrone et al. [24]	2015	BCR	106.8	4	[^18^F]F-choline	Adrenal
Zhao et al. [27]	2022	BCR	54.7	5	[^68^Ga]Ga-PSMA-11	Adrenal
Üstün et al. [25]	2024	23 patients	NA	NA	[^68^Ga]Ga-PSMA-11	Adrenal
Segal et al. [28]	2023	BCR	4.12	NA	[^18^F]F-DCFPyL	Rectal and Denonvillier fascia
Desai et al. [30]	2011	mCRPC	13.3	5	[^18^F]FDG	Pancreas
Önner et al. [31]	2023	Staging	20.1	4	[^68^Ga]Ga-PSMA-11	Liver
Kranzbühler et al. [32]	2017	BCR	30	4	[^68^Ga]Ga-PSMA-11	Liver
Chandekar et al. [33]	2023	NA	226	3	[^68^Ga]Ga-PSMA-11	Perirenal fascia
Moreau et al. [35]	2024	mPC	66	5	[^68^Ga]Ga-PSMA-11	Pericardial recess
Gandini et al. [36]	2023	mCRPC	672	5	[^18^F]F-PSMA-1007	Interatrial septum
Das et al. [37]	2022	BCR	2.5	4	[^68^Ga]Ga-PSMA-11	Right atrial
Perez et al. [38]	2019	BCR	0.275	NA	[^68^Ga]Ga-PSMA-11	Lung
McBean et al. [39]	2021	8 patients	Mean 157.7	5	[^68^Ga]Ga-PSMA-11	Brain
Ajit et al. [41]	2023	BCR	5.8	5	[^68^Ga]Ga-PSMA-11	Cerebellar
Kulkarni et al. [42]	2020	Staging	130	NA	[^68^Ga]Ga-PSMA-11	Trigeminal nerve
Al-Ibraheem et al. [43]	2024	mCRPC	44	NA	[^68^Ga]Ga-PSMA-11	Brain and muscle
Sivrikoz et al. [46]	2023	mPC	755	5	[^68^Ga]Ga-PSMA-11	Skin
Arora et al. [47]	2020	Staging	140	3	[^68^Ga]Ga-PSMA-11	Inguinal canal

BCR = biochemical recurrence; mCRPC = metastatic castration-resistant prostate cancer; mPC = metastatic prostate cancer; NA = not available.

## 4. Discussion

As a matter of fact, the conventional idea that PCa seldom metastasizes beyond the skeleton or lymph nodes has been revolutionized in recent years. The widespread utilization of PET imaging and the improvement in technology combined with the availability of new PET radiotracers has increased the detection of atypical metastatic patterns [8,49]. In clinical terms, the presence of atypical metastases may hide a malignancy with an aggressive phenotype, characterized by a biology and natural history of the disease different from PCa with typical nodal and bone metastases [50,51]. In addition, these patients may have unspecific symptoms, as well as a discrepant relationship between clinical course and serum PSA level. These features may have an impact on clinical trial eligibility, patient management, and prognosis [52,53,54].

The case reported in this paper describes a highly uncommon biochemical recurrence of PCa with testicular and, probably, lung metastases. What is even more noteworthy is that PCa metastases showed an unexpected pattern of uptake upon multi-tracer PET imaging. PSMA ligand PET is the gold standard examination in cases of biochemical recurrence of PCa [49]. However, the patient reported herein presented PCa metastases with high uptake upon [^18^F]FDG PET and absent/faint uptake upon [^68^Ga]Ga-PSMA-11 PET. Nevertheless, the histological evaluation of the right testicular lesion clearly showed a PCa origin, without evidence of neuroendocrine de-differentiation, which is known to be related to the loss of PSMA expression [55]. This case opens Pandora’s box regarding PCa tumors with low PSMA expression, which account for about 5–10% of all cases. Still, we do not know much about these PCa forms. Bauckneht et al. [56] reported that PCa patients with downregulation of the FOLH1 gene, which encodes for the PSMA protein, show a parallel increase in genes involved in glycolysis. These data reflect the clinical evidence of [^18^F]FDG-avid/PSMA-negative PCa [57]. In a recent study by Sayar and colleagues [58], evidence of at least one PSMA-negative metastasis was detected in 63% of mCRPC patients at autopsy. The authors found that MUC1 expression was inversely correlated to that of PSMA, representing a potential molecular target for PET imaging in PSMA-negative tumors. Similarly, AMACR, also known as P504S, is a mitochondrial and peroxisomal enzyme upregulated in PCa [59,60,61]. The expression of AMACR can be variable among prostate cancer histotypes; however, it maintains high sensitivity. Since the early 2000s, it has been used for prostate cancer detection when small foci of adenocarcinoma are found (in the differential diagnosis of hyperplasia versus carcinoma). Thereafter, it has also been used for robustness in cancer diagnosis along with biopsy mapping of the prostate. As a sensitive biomarker, AMACR has also been found in other neoplasms [62]. Ye et al. [63] reported that AMACR was positive in PSMA-negative tumors. This finding is consistent with the patient reported in this paper since a diffuse positivity to AMACR was noted during immunohistochemistry. Considering the evidence of simultaneous immunohistochemical expression positive for AMACR and negative for the PSMA antibody, as well as the evidence of [^18^F]FDG-avid/PSMA-negative PCa on PET imaging, AMACR is a candidate as a pioneer target for novel radiotracers in PCa [64]. Indeed, radioprobes targeting AMACR could play a complementary role to PSMA ligand PET imaging, to be reserved for patients with histological or PET evidence of absent expression of PSMA and without a history of second primary neoplasms. This possibility points toward a modern concept of multi-tracer patient-tailored imaging, similar to that recently described for [^18^F]FDG, PSMA ligands, and radiolabeled somatostatin analogs PET imaging [12]. The AMACR pathway is also involved in metabolism, such as the prestanoyl-CoA–prestanyc acid pathway. Genomic analysis focusing on the aforementioned molecules may drive additional biological knowledge. Nevertheless, data available in the literature regarding molecular expression in PSMA-negative PCa are still at an embryonal stage and there is much room for improvement. Indeed, broader knowledge of PSMA-negative PCa would improve patient selection for both diagnostic and therapeutic purposes.

The review of the literature confirmed that atypical sites of metastatic spread in PCa patients occur infrequently and occur mostly in patients with aggressive histology [54]. Most of the patients reported in the literature had an ISUP Grade Group 4 or 5. Genitourinary tract metastases may involve the testis or the penis. The detection of metastases in both localizations upon PSMA PET may be hindered by the presence of urinary contamination.

Abdominal viscera have been reported as uncommon sites of PCa metastases. Histological confirmation of metastatic involvement in abdominal viscera is difficult most of the time, as surgery is needed. However, physiological uptake upon PSMA ligand PET can be observed in the liver, jejunum, and adrenal glands. Therefore, some authors have tried to define uptake intensity cut-offs to discriminate benign vs. malignant involvement [28]. Of note, some benign conditions could simulate metastases in the abdominal viscera, i.e., liver and splenic hemangiomas, both upon PSMA ligand and [^18^F]F-choline PET [65,66,67].

Thoracic metastases occur mostly in the lungs and are usually late-stage disease localizations [68]. However, a few cases have described oligorecurrent disease limited to the lungs [39]. In patients with biochemical recurrence of PCa and lung nodules, double-tracer imaging with PSMA ligand and [^18^F]FDG PET could be considered to improve characterization and guide biopsy. Finally, cardiac, soft tissue, and brain metastases are very uncommon but easy to detect for nuclear medicine physicians due to the high TBR.

## 5. Conclusions 

Atypical metastases occur mainly in aggressive and advanced stages of PCa. Since PSMA ligand PET has been introduced into the diagnostic management of PCa, an increased number of atypical localizations has been reported in the literature. However, some subgroups of PCa may lose PSMA expression. In those cases, multi-tracer PET imaging is required and, hopefully, new radiotracers will improve detection rates. Among them, AMACR is a promising, pioneering molecular target in PSMA-negative PCa.

## Data Availability

No new data were created.
